# Cardiometabolic Changes in Sirtuin1-Heterozygous Mice on High-Fat Diet and Melatonin Supplementation

**DOI:** 10.3390/ijms25020860

**Published:** 2024-01-10

**Authors:** Gaia Favero, Igor Golic, Francesca Arnaboldi, Annalisa Cappella, Aleksandra Korac, Maria Monsalve, Alessandra Stacchiotti, Rita Rezzani

**Affiliations:** 1 Anatomy and Physiopathology Division, Department of Clinical and Experimental Sciences, University of Brescia, Viale Europa 11, 25123 Brescia, Italy; gaia.favero@unibs.it (G.F.); rita.rezzani@unibs.it (R.R.); 2 Interdipartimental University Center of Research “Adaption and Regeneration of Tissues and Organs (ARTO)”, University of Brescia, 25123 Brescia, Italy; 3 Center for Electron Microscopy, Faculty of Biology, University of Belgrade, Studentski trg 16, 11000 Belgrade, Serbia; igor.golic@bio.bg.ac.rs (I.G.); aleksandra.korac@bio.bg.ac.rs (A.K.); 4 Department of Biomedical Sciences for Health, University of Milan, Via Mangiagalli 31, 20133 Milan, Italy; francesca.arnaboldi1@unimi.it (F.A.); annalisa.cappella@unimi.it (A.C.); 5 U.O. Laboratorio di Morfologia Umana Applicata, IRCCS Policlinico San Donato, San Donato Milanese, 20097 Milan, Italy; 6 Instituto de Investigaciones Biomedicas “Alberto Sols” (CSIC-UAM), 28029 Madrid, Spain; mpmonsalve@iib.uam.es; 7 Italian Society for the Study of Orofacial Pain (Società Italiana Studio Dolore Orofacciale—SISDO), 25123 Brescia, Italy

**Keywords:** sirtuin1, melatonin, heart, epididymal adipose tissue, interscapular brown adipose tissue, mitochondria, endoplasmic reticulum stress, obesity

## Abstract

A hypercaloric fatty diet predisposes an individual to metabolic syndrome and cardiovascular complications. Sirtuin1 (SIRT1) belongs to the class III histone deacetylase family and sustains anabolism, mitochondrial biogenesis, and fat distribution. Epididymal white adipose tissue (eWAT) is involved in inflammation, whilst interscapular brown adipose tissue (iBAT) drives metabolism in obese rodents. Melatonin, a pineal indoleamine, acting as a SIRT1 modulator, may alleviate cardiometabolic damage. In the present study, we morphologically characterized the heart, eWAT, and iBAT in male heterozygous SIRT1^+/−^ mice (HET mice) on a high-fat diet (60%E lard) versus a standard rodent diet (8.5% E fat) and drinking melatonin (10 mg/kg) for 16 weeks. Wild-type (WT) male C57Bl6/J mice were similarly fed for comparison. Cardiomyocyte fibrosis and endoplasmic reticulum (ER) stress response worsened in HET mice on a high-fat diet vs. other groups. Lipid peroxidation, ER, and mitochondrial stress were assessed by 4 hydroxy-2-nonenal (4HNE), glucose-regulated protein78 (GRP78), CCAA/enhancer-binding protein homologous protein (CHOP), heat shock protein 60 (HSP60), and mitofusin2 immunostainings. Ultrastructural analysis indicated the prevalence of atypical inter-myofibrillar mitochondria with short, misaligned cristae in HET mice on a lard diet despite melatonin supplementation. Abnormal eWAT adipocytes, crown-like inflammatory structures, tumor necrosis factor alpha (TNFα), and iBAT whitening characterized HET mice on a hypercaloric fatty diet and were maintained after melatonin supply. All these data suggest that melatonin’s mechanism of action is strictly linked to full SIRT1 expression, which is required for the exhibition of effective antioxidant and anti-inflammatory properties.

## 1. Introduction

A sedentary lifestyle, dramatically reinforced by the recent COVID-19 lockdown; overweight; and obesity contribute to cardiovascular diseases and related multimorbidity, like diabetic cardiomyopathy, coronary artery disease, and cancer [1,2,3]. Indeed, when body mass index exceeds 30 kg/m^2^, the risk of cardiometabolic complications increases [4]. These events are aggravated by the aging process when chronic heart diseases may occur, decreasing life expectancy [5]. Thus, when a patient is admitted to a hospital emergency room for cardiac damage, screening for multiple metabolic adverse events is strongly recommended. Together with a sedentary lifestyle, a caloric diet rich in fat and sugars contributes to obesity in humans and rodents [6]. A hypercaloric fatty diet induces cardiac mitochondria changes, oxidative stress, calcium flux alterations, fibrosis, and inflammation, linked to insulin resistance, in rodent models of diabetes and in patients [7].

Given the strict connection between the heart and adipose tissue (AT) in the regulation of metabolism, specific adipose depots must be considered [8]. Indeed, the abdominal visceral white AT (WAT), which forms more than 95% of the adipose mass in humans, is the main target of lipid deposition and energy dissipation during high-calorie intake [9]. However, in male mice, the epididymal WAT (eWAT) is the unique depot prone to inflammation and obesogenic remodeling [10,11]. The interscapular AT depot, called brown AT (iBAT), is fundamental for combating obesity and diabetes, favoring cardiac remodeling after mild infarction in mice [12]. The main histological fingerprint of pro-inflammatory changes in AT depots is the crown-like structure (CLS), a peculiar arrangement of infiltrating macrophages around dying adipocytes [13]. So, it is important to evaluate the “quality” of AT, its pro-inflammatory acquisition, and not only its quantity to efficiently manage cardiovascular diseases [14]. Cardiovascular damage and abdominal AT remodeling are strictly related, sharing common pathogenic mechanisms, in obese patients and in rodents [15,16]. The main pathogenetic factors are endoplasmic reticulum (ER) stress, oxidative stress, and chronic inflammation [17].

In cardiomyocytes, misfolded mitochondrial proteins are deposited in the ER, and perturbation of calcium flux activates an aberrant ER reaction, called the unfolded protein response [18]. This mechanism is initiated by resident ER sensors that upregulate the transcription of master ER chaperone, glucose-regulated protein78 (GRP78), and proapoptotic C/EBP homologous protein (CHOP) [19,20]. Another chaperone, called heat shock protein 60 (HSP60), is involved in recovering mitochondrial protein folding, and its deletion in the heart induced dilated cardiomyopathy in mice [21]. So, handling proper clearance of proteins is pivotal in the heart where the ER and mitochondria are strictly associated at Z lines, where they drive calcium flux for contractility [22,23,24].

Mitochondria represent up to 45% of a single cardiomyocyte volume and are scattered in different subcellular sites and so defined as subsarcolemmal, inter-myofibrillar (IMF), and perinuclear populations [25]. Despite the forced regular presence of mitochondria in the sarcomere, their dynamism and ability to change size are essential to maintaining energetic metabolism in the heart [26,27]. Indeed, the prevalence of small mitochondria, called fission, or elongated mitochondria, called fusion, may be detrimental or beneficial for ATP production [28] and precedes reactive oxygen species production [29]. Oxidative damage in the heart triggers lipid peroxidation of mitochondrial membranes which generates toxic aldehydes like 4-hydroxynonenal (4HNE) and overt energetic failure [30].

Sirtuin1 (SIRT1), the most studied isoform of human sirtuin proteins, homologs of the Sir2 gene in yeast, is involved in epigenetic regulation and mitochondrial metabolism [31,32]. Indeed, SIRT1, acting as a lysine deacetylase via nicotinamide adenine dinucleotide on histones and non-histone proteins, controls the survival or death of cardiomyocytes in aging and diseases [33,34]. Moreover, in the heart, SIRT1 activates transcription factors able to modulate mitochondrial biogenesis [35,36]. Notably, in visceral AT, SIRT1 regulates adipogenesis, limiting obesity and glucose intolerance [37].

SIRT1-knockout (KO) mice presented developmental cardiac defects, growth retardation, and perinatal mortality [38]. Conversely, heterozygous SIRT1^+/−^ mice survived until adulthood but presented left ventricular dilatation [39]. Studies on adult cardiac-specific SIRT1-KO mice indicated a higher predisposition to adverse changes like diabetic cardiomyopathy, ER stress, and apoptosis [40,41]. By contrast, SIRT1 upregulation alleviated cardiac oxidative damage and diabetic vascular complications [42] even if excessive SIRT1 overexpression induced cardiomyopathy [43]. Similarly, mice carrying an adipocyte-specific deletion of SIRT1 developed insulin resistance and AT inflammation [44].

Melatonin (N-acetyl-5-methoxytryptamine) is an endogenous indoleamine produced by the pineal gland, with a pivotal role in the synchronization of circadian rhythms, and is able to positively influence the cardiovascular system [45] and to control energy expenditure in obesity [46]. Melatonin reaches mitochondria via specific transporters, limits mitochondrial fission, and activates mitophagy [47]. Previous studies indicate that melatonin enhances SIRT1 expression in the heart, thus reducing oxidative damage and inflammation in obese leptin-deficient mice and in diabetic mice [48,49]. Moreover, melatonin controls body weight, increases BAT depot mass, and stimulates thermogenic genes in dietary-induced obese mice [50] and in Zucker diabetic rats [51]. Compelling evidence outlines the potentiality of melatonin as a preventive or supplementary treatment in animal models of cardiometabolic disorders [52,53].

In this study, we analyzed the heart, eWAT, and interscapular BAT (iBAT) in heterozygous SIRT1^+/−^ mice (HET mice) fed a hypercaloric diet based on lard and drinking melatonin for 16 weeks. Oxidative damage, ER stress, mitochondrial chaperones, and inflammation were evaluated. Furthermore, inter-myofibrillar mitochondria were estimated to better address the role of melatonin in this particular animal model.

Our results indicated that SIRT1 haploinsufficiency in mice nullified the protective efficacy of melatonin in the heart and AT, failing to limit dietary-induced cardiometabolic damage and underlying that melatonin action is strictly linked to full SIRT1 expression.

## 2. Results

### 2.1. Sirtuin1 Content in Mouse Heart

In the heart, the amount of nuclear SIRT1 in wild-type (WT) mice on a high-fat diet (HFD) was markedly lower compared to WT mice on a standard diet (STD), but notably, supplementation of WT mice on an HFD with melatonin prevented a decrease in SIRT1. Conversely, HET mice showed a very weak nuclear SIRT1 band independently of the diet and melatonin supplementation. These observations are summarized in Supplementary Figure S1.

### 2.2. Metabolic Data and Cardiac Fibrosis

Metabolic parameters of whole-body heterozygous SIRT1 mice (HET mice) on an STD have already been reported by Planavila et al. [39]. Furthermore, plasmatic glucose measurements and indirect calorimetry in WT and HET mice on the lard-based regimen were previously indicated [54]. Considering that all parameters analyzed were similar in WT and HET mice on an STD with or without melatonin, we decided to report data of mice on a normocaloric diet as “controls” here. The body weight and eWAT weight of WT and HET mice on a standard diet (STD) or an HFD and drinking or not drinking melatonin are presented in Table 1. The body and eWAT weights were measured at the beginning of treatments (T0), in 12-week-old mice, and after 16 weeks of dietary treatment (T1), in 28-week-old mice. At the end of treatments (T1), the increment in body weight was statistically significant both in WT and HET mice (32–34% increase vs. relative littermates, *p* < 0.05). Remarkably, melatonin supplementation promoted a significant reduction in body weight (12%) in WT mice on an HFD. In contrast, HET mice on an HFD drinking melatonin presented only a limited weight loss (6%).

Similarly, eWAT weight was recorded at the end of treatments. In WT mice on a lard regimen, eWAT weight doubled in comparison with that in standard-diet-fed littermates, and melatonin promoted a weight decrease of 35%. Curiously, in HET mice, eWAT weight increased poorly after the obesogenic regimen and decreased by 20% after melatonin intake. According to Xu et al. [55], iBAT weight was not estimated due to its extremely limited amount (0.06–0.07 g) which makes the appreciation of differences inconsistent.

As weight in rodents fed an obesogenic diet is strictly dependent on age and energy dissipation, our data refer to young-adult mice (7 months old) maintained at 22 °C, a temperature lower than thermoneutrality (28 °C) [56].

Notably, even if the amounts of daily food/water intake were similar, HET mice fed an HFD demonstrated reduced locomotor activity and oxygen consumption, at night, that might be the reason for abnormal body weight and eWAT depot parameters [54].

**Figure 1:**
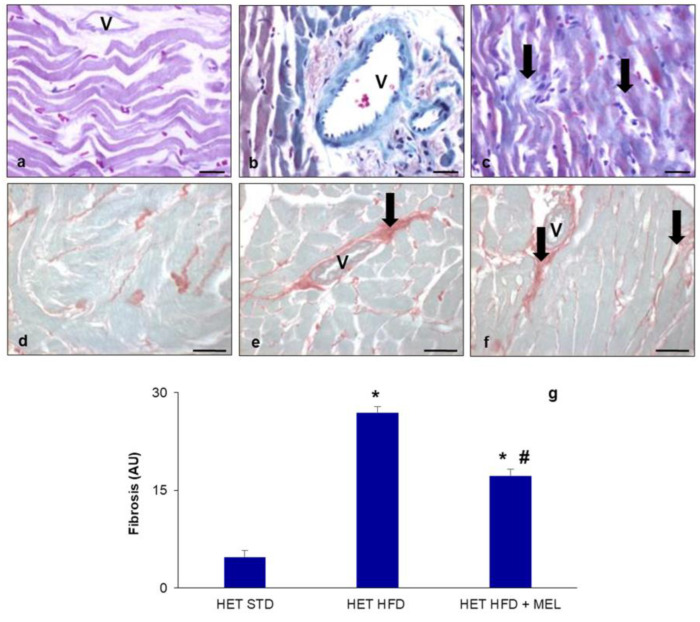
Heart fibrosis. Representative photomicrographs showing perivascular and interstitial collagen deposition (arrows) in the left ventricles of (**a**,**d**) HET mice on standard diet, (**b**,**e**) HET mice on HFD, (**c**,**f**) HET mice on HFD plus melatonin. Masson’s staining (**a**–**c**) and Sirius Red staining (**d**–**f**). Original magnification: 400× (**a**–**c**) and 200× (**d**–**f**). Bars = 20 µm (**a**–**c**) and 50 µm (**d**–**f**). Morphometrical estimation of total heart fibrosis (**g**). * *p* < 0.05 vs. HET mice on STD; # *p* < 0.05 vs. HET mice on HFD. AU: arbitrary units; HET: SIRT1^+/−^; HFD: high-fat diet (TD 03584-lard 35%); MEL: melatonin; STD: standard maintenance diet; V: coronary vessel.

Then, we focused on the heart, performing histological analysis of fibrosis, a quite common sign of obesity. Fibrosis, evident as blue color in Masson’s staining (Figure 1a–c) and as red color in Sirius Red staining (Figure 1d–f), was absent in HET mice on a standard diet (Figure 1a,d) but evident in HET mice fed an HFD for 16 weeks, which presented significant (*p* < 0.05) perivascular (coronary vessels) and interstitial fibrosis (Figure 1b,e). This morphological feature was still maintained in the HFD plus melatonin group, mainly in the perivascular area (Figure 1c,f), corresponding to a total fibrosis index greater than 15% (Figure 1g).

### 2.3. Heart Lipid Peroxidation and Endoplasmic Reticulum Stress

**Figure 2:**
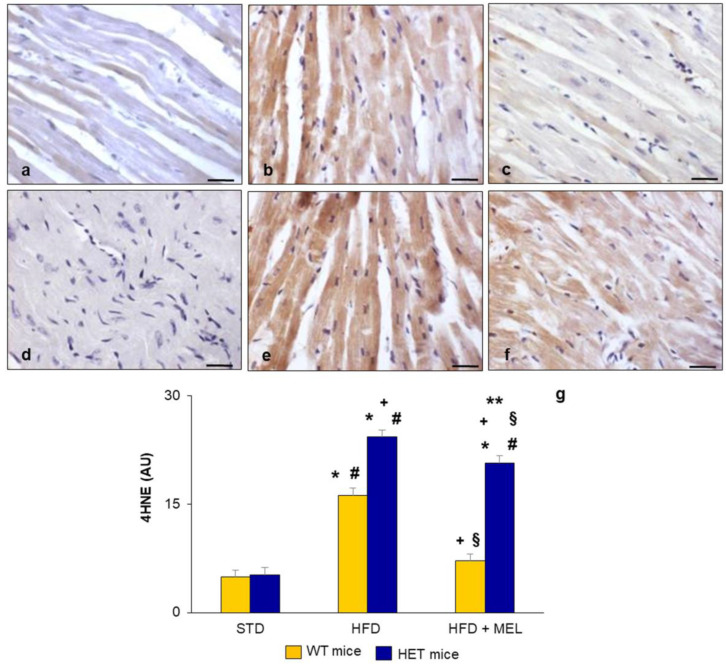
Heart lipid peroxidation. Representative photomicrographs of ventricular cardiomyocyte 4HNE immunostaining in (**a**) WT mice on standard diet, (**b**) WT mice on HFD, (**c**) WT mice on HFD plus melatonin, (**d**) HET mice on standard diet, (**e**) HET mice on HFD, (**f**) HET mice on HFD plus melatonin. Original magnification: 400×, bars = 20 µm. Quantitative analysis of 4HNE immunopositivity indicated the maintenance of toxic lipid peroxidation products in HET mice (**g**). * *p* < 0.05 vs. WT mice on STD; # *p* < 0.05 vs. HET mice on STD; + *p* < 0.05 vs. WT mice on HFD; § *p* < 0.05 vs. HET mice on HFD; ** *p* < 0.05 vs. WT mice on HFD plus melatonin. AU: arbitrary units; HET: SIRT1^+/−^; HFD: high-fat diet (TD 03584-lard 35%); MEL: melatonin; STD: standard maintenance diet.

Abnormal body weight and fat depots, together with cardiac fibrotic changes, suggested the onset of metabolic damage, starting early in HET mice on a lard diet for 16 weeks. For this reason, in left ventricular cardiomyocytes, we analyzed the presence of 4 hydroxy-2-nonenal (4HNE), a lipid peroxidation marker associated with oxidative damage. Indeed, toxic lipid peroxidation products are produced in cardiac mitochondria when reactive oxygen species attack unsaturated lipid membranes, and they represent an index of inflammation and oxidative damage [57]. The immunohistochemical analysis of 4HNE indicated its absence in WT and HET mice placed on a standard diet (Figure 2a,d) but a strong brown signal in WT and HET mice on an HFD (Figure 2b,e), reduced only in WT mice after melatonin intake (Figure 2c). Conversely, in HET mice on an HFD plus melatonin, moderate 4HNE immunostaining was still detected (Figure 2f), indicating the persistence of lipid peroxidation in this group. The morphometrical quantification of 4HNE immunostaining is plotted in Figure 2g.

Considering the strong relationship between lipid peroxidation, SIRT1, and ER stress in the heart, we analyzed the presence of ER stress in ventricular cardiomyocytes. GRP78, the master regulator of the ER stress response, was localized using immunohistochemistry in WT and HET mice under different diets [40].

**Figure 3:**
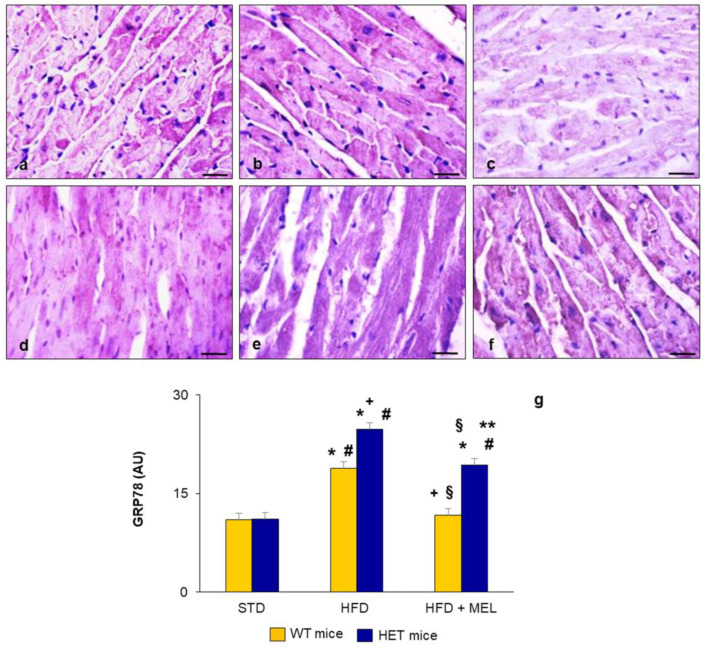
Heart endoplasmic reticulum stress. Representative photomicrographs of GRP78 immunostaining showing the extent of ER stress response in brown (**a**) WT mice on standard diet, (**b**) WT mice on HFD, (**c**) WT mice on HFD plus melatonin, (**d**) HET mice on standard diet, (**e**) HET mice on HFD, (**f**) HET mice on HFD plus melatonin. Original magnification: 400×, bars = 20 µm. (**g**) Quantitative analysis of GRP78 immunopositivity. * *p* < 0.05 vs. WT mice on STD; # *p* < 0.05 vs. HET mice on STD; + *p* < 0.05 vs. WT mice on HFD; § *p* < 0.05 vs. HET mice on HFD; ** *p* < 0.05 vs. WT mice on HFD plus melatonin. AU: arbitrary units; HET: SIRT1^+/−^; HFD: high-fat diet (TD 03584-lard 35%); MEL: melatonin; STD: standard maintenance diet.

A faint GRP78 signal was localized in the heart of WT and HET mice fed a standard maintenance diet (Figure 3a,d). GRP78 distribution was limited to a few cardiomyocytes in WT mice on an HFD (Figure 3b) and almost absent in WT mice drinking melatonin (Figure 3c). In contrast, GRP78 was significantly enhanced in HET mice on an obesogenic diet (Figure 3e) and was still moderate after melatonin supplementation (Figure 3f). All these findings suggest the occurrence of sustained ER stress in the HET group fed a hypercaloric diet. The morphometrical quantification of GRP78 immunostaining is plotted in Figure 3g.

To further assess the involvement of ER-driven proapoptotic events in the heart, we analyzed nuclear CHOP presence in cardiomyocytes. The immune reaction was undetectable in WT mice (Supplementary Figure S2a–c) and HET mice (Supplementary Figure S2d–f) on a maintenance normocaloric diet but evident under an HFD. Nuclear CHOP immunostaining was absent in WT mice on an HFD plus melatonin (Supplementary Figure S2c) but persisted in HET mice on an HFD plus melatonin (Supplementary Figure S2f).

### 2.4. Ultrastructural Analysis of Heart Mitochondria

**Figure 4:**
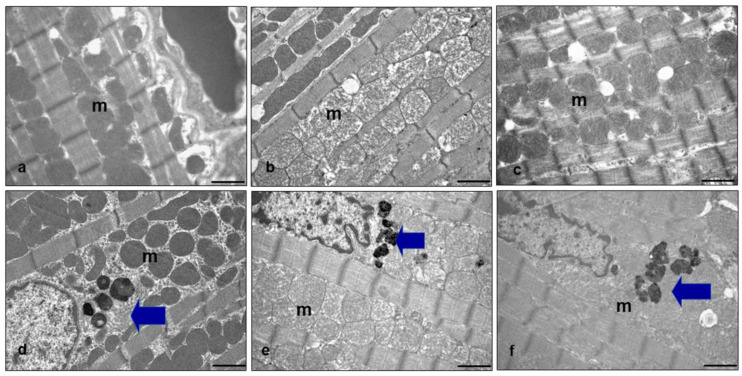
Ultrastructural analysis of heart mitochondria. Representative transmission electron microscopy heart photomicrographs of (**a**) WT mice on standard diet, (**b**) WT mice on HFD, (**c**) WT mice on HFD plus melatonin, (**d**) HET mice on standard diet, (**e**) HET mice on HFD, (**f**) HET mice on HFD plus melatonin. Note the differences in size, density and cristae composition of inter-myofibrillary mitochondria (m) under different dietary regimens. Blue arrows indicate lipofuscins. Bars = 1 µm.

To better assess oxidative damage induced by a fatty diet, we performed an ultrastructural analysis of inter-myofibrillary mitochondria (IFM) in the ventricular cardiomyocytes of each experimental group. This is because IFM are mainly detected in metabolic remodeling and damaged in heart failure [58]. In WT mice on an STD, round mitochondria were regularly juxtaposed with sarcomeres (Figure 4a). They were enlarged and lost cristae in WT mice on an HFD (Figure 4b) and recovered in WT mice on an HFD drinking melatonin (Figure 4c). In HET mice on an STD, IFM presented a regular distribution and dense inner matrix, and large perinuclear lipofuscin deposits were evident (Figure 4d). In HET mice on an HFD, IFM became heterogeneous, showing poor cristae (Figure 4e). Finally, in HET mice on an HFD plus melatonin, abnormal mitochondria with a pale inner matrix and devoid of cristae or with very altered cristae were evident (Figure 4f). In HET mice fed a lard diet with or without melatonin, abundant perinuclear lipofuscins were still observed.

**Figure 5:**
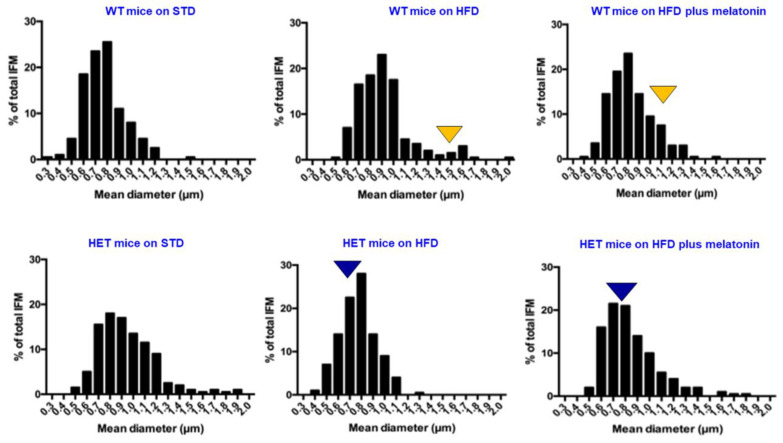
Morphometry of heart inter-myofibrillar mitochondria. Morphometric analysis of mean inter-myofibrillary mitochondrion diameter using transmission electron microscopy. Yellow arrowheads indicate enlarged mitochondria in WT mice; blue arrowheads indicate the most frequent small-sized mitochondria in HET mice. HET: SIRT1^+/−^; HFD: high-fat diet (TD 03584-lard 35%); STD: standard maintenance diet.

To best corroborate the response of IFM to different diets in WT vs. HET mice, we performed a morphometric analysis of mitochondrion size using transmission electron microscopy, as indicated in Figure 5.

The mean mitochondrion Feret’s diameter was plotted, according to classes ranging from 0.5 microns to 1.2 microns in WT mice and from 0.5 microns to 1.5 microns in HET mice on an STD. Under the lard hypercaloric regimen, the mean mitochondrion diameter exceeded 1 micron (25% frequency) in WT mice, indicating the presence of “mega-mitochondria”. Conversely, in HET mice on an HFD, the mitochondrion diameter was shorter, about 0.8 microns (about 30% frequency). After melatonin intake in WT mice on an HFD, most diameters decreased to 0.7–0.8 microns (60% frequency). In HET mice on an HFD, mitochondrion diameter was maintained at 0.6–0.8 microns (55% frequency), with or without melatonin supplementation.

To further confirm the presence of abnormally enlarged mitochondria in WT mice on an HFD compared with other experimental groups, we evaluated mitofusin2 (Mfn2), a marker of elongated mitochondria. Mfn2 immunostaining was strong in the cardiomyocytes of WT mice on a standard diet (Supplementary Figure S3a), was reduced in WT mice on a lard diet (Supplementary Figure S3b), and was restored after melatonin supplementation (Supplementary Figure S3c). All these findings suggest an adaptation of mitochondrion size only in WT mice triggered by different energetic surpluses, and the inability of mitochondria to change size in HET mice.

### 2.5. Heart Heat Shock Protein60 Expression

Abnormal oxygen consumption, ATP, and reactive oxygen species production in heart mitochondria are sensed by the mitochondrial chaperone HSP60 [59]. Indeed, HSP60 is present in the mitochondrial matrix, and it is greatly involved in adaptive remodeling in response to energetic challenges in cardiomyocytes [21].

**Figure 6:**
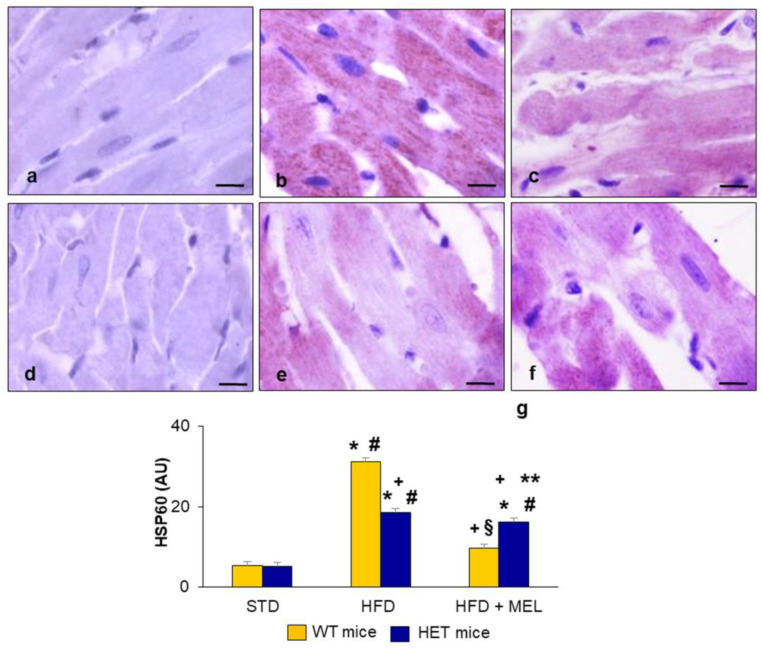
Heart heat shock protein60 expression. Representative photomicrographs of brown HSP60 immunostaining in (**a**) WT mice on standard diet, (**b**) WT mice on HFD, (**c**) WT mice on HFD plus melatonin, (**d**) HET mice on standard diet, (**e**) HET mice on HFD, (**f**) HET mice on HFD plus melatonin. Original magnification: 1000×, bars = 10 µm. (**g**) Quantitative analysis of HSP60 immunopositivity. * *p* < 0.05 vs. WT mice on STD; # *p* < 0.05 vs. HET mice on STD; + *p* < 0.05 vs. WT mice on HFD; § *p* < 0.05 vs. HET mice on HFD; ** *p* < 0.05 vs. WT mice on HFD plus melatonin. AU: arbitrary units; HET: SIRT1^+/−^; HFD: high-fat diet (TD 03584-lard 35%); MEL: melatonin; STD: standard maintenance diet; WT: wild type.

In the present study, we assessed the presence of HSP60 as a cardioprotective marker in different experimental groups (Figure 6). In WT mice (Figure 6a) and HET mice (Figure 6d) on a standard diet, the HSP60 signal was faint in cardiomyocytes. Remarkably, in WT mice on an HFD, HSP60 brown immunostaining was strong and granular in the cytoplasm (Figure 6b) but decreased after melatonin supplementation (Figure 6c). In HET mice on an HFD (Figure 6e), HSP60 staining was scattered and moderate and did not change after melatonin intake (Figure 6f). The quantitation of HSP60 is reported in Figure 6g. These data confirmed the inability of cardiac IFM in HET mice on an HFD to adapt to greater energy consumption and the hypercaloric intake provided by the lard-based diet.

### 2.6. Epididymal Adipose Tissue and Brown Adipose Tissue Evaluation

Given the close relationship between anatomic localization of AT depots, inflammation, and cardiovascular metabolism in obesity, we analyzed eWAT and iBAT in response to different diets in WT and HET mice.

Considering the anatomic variability of three different eWAT zones, to better assess the extent of inflammation, we performed hematoxylin–eosin staining in rostral eWAT according to Altintas et al. [60].

**Figure 7:**
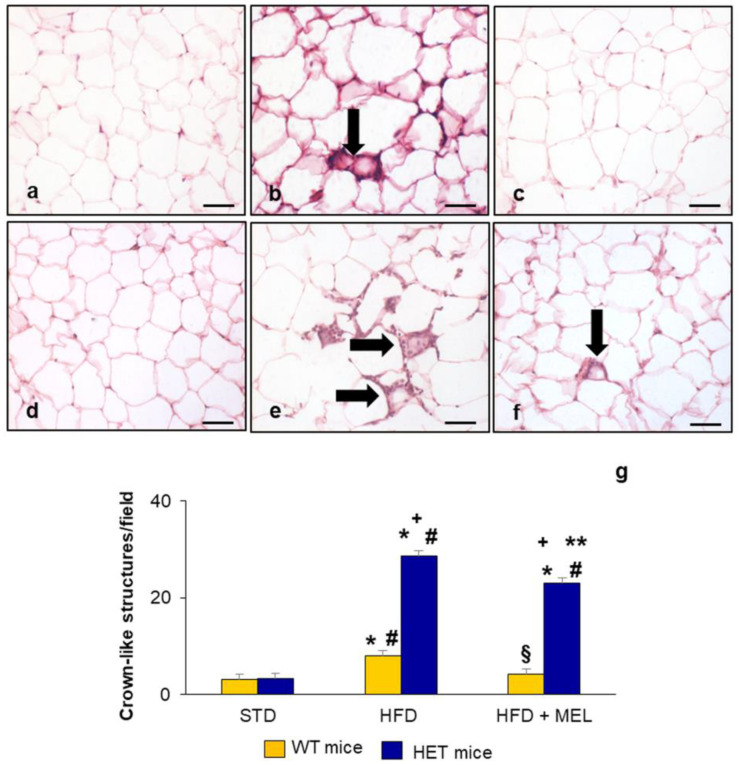
Epididymal adipose tissue crown-like structure analysis. Representative hematoxylin–eosin-stained eWAT photomicrographs: (**a**) WT mice on standard diet, (**b**) WT mice on HFD, (**c**) WT mice on HFD plus melatonin, (**d**) HET mice on standard diet, (**e**) HET mice on HFD, and (**f**) HET mice on HFD plus melatonin. The black arrows indicate the crown-like structures. Original magnification: 400×, bars = 20 µm. (**g**) Quantitative analysis of crown-like structures in epididymal adipose tissue. * *p* < 0.05 vs. WT mice on STD; # *p* < 0.05 vs. HET mice on STD; + *p* < 0.05 vs. WT mice on HFD; § *p* < 0.05 vs. HET mice on HFD; ** *p* < 0.05 vs. WT mice on HFD plus melatonin.

Firstly, in eWAT, we quantified crown-like structures (CLSs), composed of the strict association between macrophages/mastocytes and adipocytes. WT mice on an STD did not present any CLSs (Figure 7a), whereas few CLSs were found in WT mice on an HFD (Figure 7b), and the number of CLSs was reduced in WT mice on an HFD plus melatonin (Figure 7c). In HET mice on an HFD, CLS density increased to more than 10-fold versus HET mice on an STD and persisted, even at a lower number, after melatonin intake (Figure 7d–f). The quantification of CLSs in eWAT is plotted in Figure 7g.

**Figure 8:**
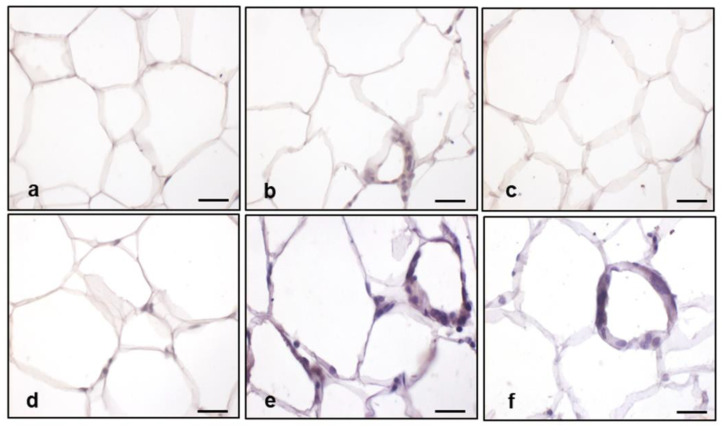
Epididymal adipose tissue tumor necrosis factor α analysis. Representative photomicrographs of TNFα immunostaining: (**a**) WT mice on STD, (**b**) WT mice on HFD, (**c**) WT mice on HFD plus melatonin, (**d**) HET mice on STD, (**e**) HET mice on HFD, and (**f**) HET mice on HFD plus melatonin. Original magnification: 400×, bars = 20 µm.

To further confirm the extent of inflammation in eWAT, we detected the presence of the cytokine tumor necrosis factor alpha (TNFα) using immunohistochemistry. TNFα immunostaining was barely detectable in WT mice on an STD (Figure 8a) and HET mice on an STD (Figure 8d), faint in WT mice on an HFD (Figure 8b), and undetectable in WT mice on an HFD plus melatonin (Figure 8c). In contrast, a strong brown TNFα immunostaining was associated with CLSs in HET mice on an HFD (Figure 8e) and was still evident in HET mice on an HFD plus melatonin (Figure 8f).

iBAT histology was multilocular in WT mice with any dietary treatment, while on the contrary, iBAT histology was predominantly unilocular, a sign of “whitening”, in HET mice on an HFD and after melatonin intake. Indeed, in these two last experimental groups, the adipocytes were full of large lipid droplets which gave them a unilocular appearance. This finding was corroborated by the estimation of nuclear density per area of adipocytes. In HET mice on an HFD, the number of nuclei was reduced by 70% compared to HET mice on an STD and by 45% compared to HET mice on an HFD plus melatonin, corresponding to 8 and 12 nuclei per field compared to 20 nuclei per field in mice on an STD (Supplementary Figure S4).

## 3. Discussion

Heart failure is a complex multifactorial adverse event that occurs because of hypertension, reduced cardiorespiratory fitness, chronic inflammation, and energetic dysfunctions [61]. The maintenance of a regular body weight starting from childhood is highly recommended to limit the risk of the onset of cardiac damage in adulthood and senescence [62]. Despite recent advances in novel gene therapy, pharmacological treatments of heart failure are not resolutive, and dietary supplementation with nutraceutical compounds is strongly recommended [22].

Melatonin, a pineal indoleamine, has been successfully added to diets in animal models of obesity to limit overweight, alleviating metabolic damage and UPR response in a dose-dependent manner [63]. Moreover, low plasma melatonin levels were detected in patients with hypertension and dilated cardiomyopathy [64,65].

A Western diet, rich in sugar and fat, affects cardiac metabolism in rodents depending on species, duration of treatments, and dietary formulation [66], and adverse effects are aggravated in SIRT1-insufficient mice [67].

Our group previously reported that HET mice on a lard-based diet with or without melatonin for 16 weeks presented inflammation, steatosis, and mitochondrial changes at the liver level [54].

In this study focused on the heart and different adipose depots, melatonin could not alleviate cardiac fibrosis, mitochondrial damage, and pro-inflammatory status in HET mice on a similar hypercaloric diet for 16 weeks. Similar adverse cardiac changes and inflammation were reported in HET mice placed on a corn-oil-based high-fat diet for a shorter time, only 5 weeks [68]. However, the proportion of saturated vs. unsaturated fatty acids in the diet variably impacts mitochondrial membrane lipids, blocking SIRT1 activity in cardiomyocytes [69]. Furthermore, full SIRT1 deficiency in the heart aggravates mitochondrial oxidative ability and induces fibrosis in mice under pressure overload [70].

Perivascular cardiac fibrosis and mitochondrial abnormalities, both in size and cristae, in HET mice decreased only partially after melatonin supplementation. In contrast, fibrosis was undetectable in WT mice on a lard-based diet for 16 weeks. This finding is because more prolonged dietary treatment, for almost 30 weeks, is necessary to obtain overt cardiac remodeling in obese mice [71,72]. However, a prolonged lard diet for over six months did not induce a different respiratory ratio in isolated mitochondria in the heart of C57BL6 mice [73].

Conversely, HET mice on an HFD with or without melatonin for 16 weeks presented exacerbated lipid peroxidation, mitochondrial oxidative damage, and ER stress indicated by 4HNE, GRP78, and CHOP immunostainings. The persistence of oxidative lipid peroxidation products, like 4HNE, and the activation of mitochondrial/ER chaperones are early factors of cardiomyopathy and ischemic damage, preceding overt heart failure [8,74]. In contrast, the same markers deeply decreased in the heart of WT mice placed on an HFD plus melatonin.

In addition, the HSP60 chaperone detected in ventricular cardiomyocytes, in WT mice on an HFD, might represent an adaptive reaction to counteract mitochondrial proteins misfolding, which is then recovered due to melatonin consumption [75]. On the contrary, a faint HSP60 signal in HET mice on an HFD with or without melatonin suggested a mitochondrial inability to adapt to the energetic surplus provided by lard.

Similarly, proapoptotic CHOP was detected in the cardiomyocyte nuclei of HET mice on an HFD with or without melatonin intake [76]. This last finding well correlates with the role of SIRT1 in directly deacetylating eIF2a/PERK, one of the three branches of ER stress, thus limiting CHOP-induced apoptosis [41]. Recently, in lipopolysaccharide-stimulated chondrocytes, ER-stress-mediated apoptosis via CHOP was antagonized by melatonin via SIRT1 expression [27]. A similar trend was reported by Yang et al. [77] in H9C2 cardiomyocytes treated with palmitic acid, which negatively impacts SIRT1 activity. Both full expression and activity of SIRT1 greatly affect the mitochondrial role, ER stress, and cell death in different cellular types and may be regulated by melatonin.

An interesting morphologic finding reported in this study is the perinuclear deposition of lipofuscins in the heart of adult HET mice, independently of the diet, suggesting the inability to dismantle aberrant proteins and lipids [78]. Indeed, lipofuscins, a well-known hallmark of senescence, are composed of oxidized proteins, lipids, and metals [79]. Even if we did not analyze autophagy in the heart here, in the liver of HET mice, we documented inconsistent autophagy already in basal conditions [54]. Furthermore, accelerated senescence has been reported in cardiac hypertrophy in mice defective in mitochondrial shaping proteins [80], and lipofuscins correlate with mitochondria swelling in HET mice, as indicated by Alam et al. [26].

Another peculiar result reported here is the prevalence of small, round mitochondria in HET mice regardless of the diet, in line with low Mfn2 expression. Notably, Mfn2, a marker of fused and elongated mitochondria, was moderate in WT mice on an STD, decreased in WT mice on an HFD, and restored in WT mice on an HFD plus melatonin. These findings might be the consequence of full SIRT1 expression in WT mice, and its ability to deacetylate Mfn2, contributing to proper mitochondrial adaptation and dynamism [80].

**Figure 9:**
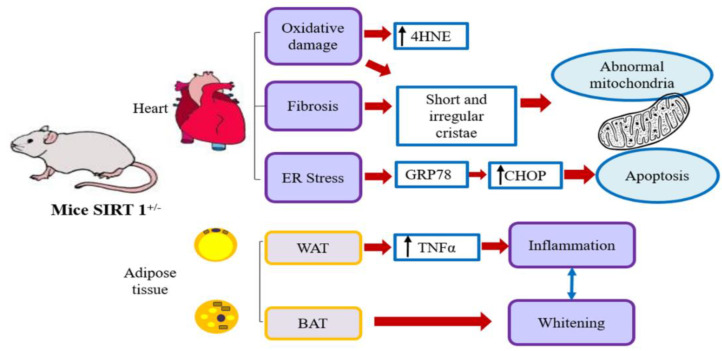
HET heart and adipose tissue alterations. Schematic representation of heart and adipose tissue depot alterations observed in HET mice on a hypercaloric lard-based diet. 4HNE: 4-hydroxynonenal; BAT: brown adipose tissue; GRP78: glucose-regulated protein 78; CHOP: CCAA/enhancer-binding protein homologous protein; TNFα: tumor necrosis factor alpha; WAT: white adipose tissue.

Regarding eWAT analysis, a noteworthy finding addressed here is the inflammatory reaction in HET mice on a lard diet with or without melatonin. Conversely, in WT mice on an HFD mice plus melatonin, no local inflammation was detectable. The inflammatory response in rostral eWAT was linked to high CLS density and well correlated with SIRT1 heterozygosity and insulin resistance reported in HET mice [81]. Intriguingly, eWAT was the unique site of synthesis of plasmatic SIRT1, necessary for lipolysis and regulation of glucose [82]. SIRT1 haploinsufficiency probably affected adipocyte metabolism in mice placed on an obesogenic diet. Our group previously reported that melatonin supplementation for 8 weeks, at a higher dose (100 mg/kg/day), reduced inflammation and hypertrophy in abdominal visceral AT in leptin-deficient genetically obese mice [83]. Furthermore, iBAT “whitening”, morphologically characterized by the presence of large unilocular adipocytes and reduced nuclear density, was evident in HET mice on a lard diet and maintained in mice drinking melatonin, housed at 22 °C, below thermoneutrality. This last finding agrees with reduced energy expenditure registered in HET mice and with mitochondrial degeneration reported in iBAT by Xu et al. [55]. In conclusion, adult HET mice on a hypercaloric lard-based diet displayed morphologic and metabolic changes in the heart and AT depots, as presented in Figure 9. The data reported in the present study support the fundamental interdependence between full SIRT1 expression and melatonin allowing the latter to exert its antioxidant and anti-inflammatory role in obesity.

However, some limitations occurred in this study. Firstly, this was intentionally a morphological study, considering the large body of evidence in the literature on molecular characterization of SIRT1-deficient mice (KO or heterozygous) with dietary-induced obesity and in vitro cardiomyocytes. Second, different sirtuin isoforms, mainly SIRT3 and SIRT6, might be considered as mitochondrial markers of heart failure and require future dedicated studies. Third, we focused here on male adult mice, but sex differences associated with obesity, estrogen levels, insulin resistance, and cardiovascular damage should be evaluated in females. The gender-dependent point might be addressed because the spatial organization of mitochondrial subpopulations is different in male vs. female hearts [84]. Finally, considering the different light/day biorhythms in mice vs. humans, more clinical studies are mandatory to highlight the strong interconnection between melatonin and SIRT1 expression in cardiac obesity in male and female patients at different lifespan periods.

## 4. Materials and Methods

### 4.1. Animal Model and Dietary Treatments

In this study, we adopted well-characterized SIRT1^+/−^ heterozygous mice (HET mice) obtained after five generations of breeding on a C57Bl/6J background at CSIC-UAM. All treatments were carried out in compliance with the European Community Commission directive guidelines (2020/63/UE) and approved by the local CSIC Ethical Committee (code D.N.I. 50840973W). Male HET mice and wild-type (WT) littermates, at 12 weeks of age, were randomly divided into the following experimental groups (6–10 mice/group): (1) WT mice fed with a standard maintenance diet (total energy 315 kcal/100 g); (2) WT mice fed with a standard diet and orally treated with melatonin; (3) HET mice fed a standard diet; (4) HET mice fed a standard diet and orally treated with melatonin; (5) WT mice fed with an HFD (total energy 540 kcal/100 g); (6) WT mice fed with an HFD and orally treated with melatonin; (7) HET mice fed an HFD; and (8) HET mice fed an HFD and orally treated with melatonin. According to European Regulation 86/609 EEC based on the 3Rs principle of animal welfare, the number of mice on a standard diet and on a standard diet plus melatonin supplementation was limited (n = 6/experimental group). Melatonin (Melapure™ kindly provided by Flamma S.p.A., Chignolo d’Isola, Bergamo, Italy) was dissolved in 1% ethanol and diluted in drinking water to yield a final dose of 10 mg/kg body weight/day. The drinking bottles containing melatonin solution were wrapped with aluminum foil to maintain dark conditions, made fresh, and replaced twice every week. The dietary formulations adopted in this study are presented in Supplementary Table S1. More details on the HFD formulation are reported by Mao et al. [85].

At the end of the treatment period, all experimental animals were sacrificed at 28 weeks of age by euthanasia in the morning starting from 10:00 a.m. The heart, eWAT, and iBAT were collected and adequately processed for morphological, immunohistochemical, and Western blotting analyses.

All samples were fixed in 4% buffered paraformaldehyde for 24 h, paraffin-embedded, and then sectioned using a microtome (5 µm thick). A small part of the ventricular heart sample of each experimental animal was adequately proceeded for transmission electron microscopy evaluation or for SIRT1 Western blotting evaluation, as further described in detail.

### 4.2. Morphological and Morphometrical Analysis

AT paraffin alternate sections were deparaffinized, rehydrated, and stained with hematoxylin–eosin. Nuclear density in iBAT was estimated in hematoxylin–eosin-stained sections digitally imaged and calculated as the total number referring to each digital frame corresponding to 0.04 mm^2^ (light microscope magnification of 400×) [86]. Fifteen fields were randomly analyzed for each experimental animal. The CLSs in eWAT were counted on ten randomly selected fields (light microscope final magnification of 200×) for five non-consecutive hematoxylin–eosin-stained sections per experimental animal using an image analysis program (Image Pro Premier 9.1, Media Cybernetics, Rockville, MD, USA).

To assess fibrosis, heart paraffin sections were stained with Masson Trichrome and Sirius Red stainings, following standard protocols. The percentage of fibrosis from both Masson Trichrome- and Sirius Red-stained sections (observed in blue and red color, respectively) was evaluated using the same image analysis program and expressed in arbitrary units (AU).

All the morphometrical evaluations were performed by two observers blinded to the experimental groups, and it was assumed that the morphological evaluations were correct if there were no statistically different values between the investigators.

### 4.3. Immunohistochemical Analysis

Alternate heart and eWAT paraffin sections were deparaffinized, rehydrated, and then incubated with 3% hydrogen peroxide for 30 min to block endogenous peroxidase activity. The sections were then incubated with specific normal serum for 60 min at room temperature, and then, heart sections with the primary antibodies polyclonal anti-rabbit 4HNE (diluted 1:400; Abcam, Cambridge, UK), polyclonal anti-rabbit GRP78 (diluted 1:250; Abcam, Cambridge, UK), monoclonal anti-mouse HSP60 (diluted 1:200; StressGen, Enzo Life Science, Lausen, Switzerland), polyclonal anti-goat CHOP (diluted 1:50; Santa Cruz Biotechnology Inc., Dallas, TX, USA), and polyclonal anti-rabbit mitofusin2 antibody (1:500; Abcam; Cambridge, UK) and AT sections were incubated with monoclonal anti-mouse TNFα (diluted 1:200; Santa Cruz Biotechnology Inc., Dallas, TX, USA) for 1 h at room temperature and overnight at +4 °C. Both heart and AT sections were sequentially incubated with specific biotinylated immunoglobulins, avidin–biotin–peroxidase complex (Vector Labs, Burlingame, CA, USA), and 0.05% 3-3-diaminobenzidine tetrahydrochloride (DAB; Sigma Aldrich, St. Louis, MO, USA) and finally counterstained with hematoxylin [87]. A negative control for each immunohistochemical staining was produced by omitting the primary antibody in the presence of isotype-matched IgG.

The sections were then observed with an optical microscope (Olympus, Hamburg, Germany) by two observers blinded to the treatments. Ten randomly selected fields for five non-consecutive sections for each experimental animal were analyzed, and the immunopositivity for each primary antibody was evaluated using an image analysis program (Image Pro Premier 9.1, Media Cybernetics, Rockville, MD, USA) and expressed in arbitrary units (AU). We assumed that the evaluations were correct if there were no statistically different values between the two investigators.

### 4.4. Transmission Electron Microscopy

A small part of the heart of each experimental animal was fragmented in small pieces, fixed in 2.5% glutaraldehyde and cacodylate buffer 0.1 M for 3 h, and then post-fixed in 2% osmium tetroxide in the same buffer for 1 h at +4 °C. Then the samples were dehydrated in progressive ethanol concentrations and propylene oxide and embedded in an Epon 812 mixture, as previously described [49]. Thin sections (80 nm) were collected on copper grids, double stained in uranyl acetate and lead citrate, and observed under a transmission electron microscope (Philips CM12, FEI Company, Eindhoven, The Netherlands) set at 80 kV.

Feret’s diameter, i.e., the longest distance between any two points within a given mitochondrion, was analyzed in the IFM population. Mean values were obtained using Image Processing and Analyses in JAVA, NIH, Bethesda, MD, USA and different classes of mitochondria were plotted by diameter. Two hundred IFM were randomly estimated in four mice/group at a final magnification of 13,000×.

### 4.5. Western Blotting

A part of each heart (including atria and ventricles) was removed and immediately frozen in liquid nitrogen and stored at −80° C until the Western blotting assay was performed.

According to the manufacturer’s instructions (Nuclear and Cytoplasmic Extraction kit, Thermo Scientific, Rockford, IL, USA), nuclear and cytoplasmatic extraction was performed. The protein concentration of the nuclear extract was determined using a Pierce BCA assay kit (Thermofisher, Rockford, IL, USA). Equal amounts of proteins (40 μg) were loaded into 10% SDS polyacrylamide gels and subjected to electrophoresis. The separated proteins were transferred to nitrocellulose membranes, and then the membranes were blocked with 1% bovine serum albumin solution for 1 h, followed by overnight incubation at 4 °C with the following primary antibodies: mouse monoclonal SIRT1 (diluted 1:1000; Abcam, Cambridge, United Kingdom) and mouse monoclonal β-actin antibody (diluted 1:5000; Sigma-Aldrich, St. Louis, MO, USA). After washing with tris-buffered saline w/tween-20, the blots were incubated with biotinylated specific immunoglobulins (Vector Labs., Burlingame, CA, USA) for 1 h at room temperature. Subsequently, the membranes were incubated in avidin–biotin–peroxidase complex (Vector Labs., Burlingame, CA, USA). The reaction products were visualized using 0.05% diaminobenzidine plus 0.03% hydrogen peroxide (Sigma, St. Louis, MO, USA) as chromogen [88].

### 4.6. Statistical Analysis

All results are indicated as mean ± standard deviation (SD). Analysis of statistical significance was performed using one-way or two-way analysis of variance (ANOVA) corrected using the Bonferroni test to determine differences between single treatment groups. *p* < 0.05 was set as a significant value.

## Figures and Tables

**Table 1:** Metabolic data: body weight and eWAT weight.

	STD (n = 6)	HFD (n = 10)	HFD + MEL (n = 10)
WT body weight—T0 (g)	26.35 ± 0.8	27.58 ± 0.7	26.49 ± 0.9
WT body weight—T1 (g)	29.09 ± 0.9	42.35 ± 1.1 ^A,C^	34.05 ± 0.7 ^B^
WT body weight gain (g)	2.74	14.77	7.56
HET body weight—T0 (g)	27.03 ± 0.8	27.34 ± 0.9	27.65 ± 0.8
HET body weight—T1 (g)	30.06 ± 0.7	40.42 ± 1.0 ^A^	38.10 ± 0.6 ^A^
HET body weight gain (g)	3.03	13.08	10.45
WT eWAT weight—T0 (g)	0.82 ± 0.04	1.12 ± 0.02 ^A,C^	0.93 ± 0.03 ^A,B^
HET eWAT weight—T1 (g)	0.78 ± 0.02	0.70± 0.02 ^A^	0.68 ± 0.04 ^A^

Values are means ± standard deviation. eWAT: epididymal adipose tissue; HET: SIRT1^+/−^; STD: standard maintenance diet; HFD: high-fat diet (TD 03584-lard 35%); MEL: melatonin; WT: wild type. n = number of mice. A, *p* < 0.05 vs. STD; B, *p* < 0.05 vs. HFD; C, *p* < 0.05 vs. HFD plus melatonin.

## Data Availability

The data presented in this study are available from the corresponding author upon reasonable request.

## Supplementary Materials

The supporting information can be downloaded at: https://www.mdpi.com/article/10.3390/ijms25020860/s1.
