# Supplementary material for: Cardiometabolic Changes in Sirtuin1-Heterozygous Mice on High-Fat Diet and Melatonin Supplementation
Source: Int J Mol Sci. 2024 Jan 10;25(2):860. doi: 10.3390/ijms25020860 (PMC10815439; doi:10.3390/ijms25020860)
Supplement: Supplementary file 1 [file ijms-25-00860-s001.zip › Supplementary Figures.docx]

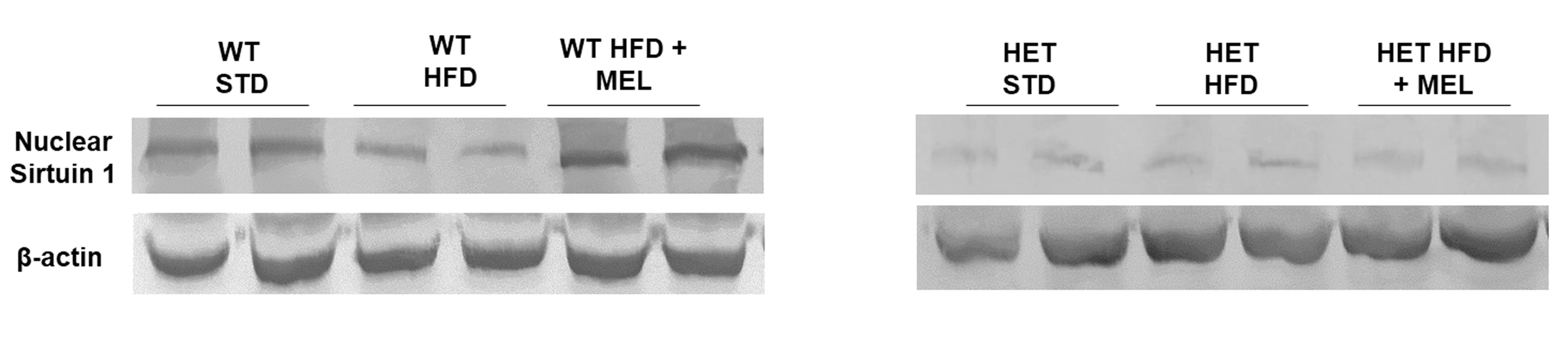


**Supplementary Figure S1**: *Nuclear sirtuin1 protein level at heart level*. Representative Western blotting for nuclear sirtuin1 protein level in WT mice on standard diet, WT mice on HFD mice, WT mice on HFD plus melatonin, HET mice on standard diet, HET mice on HFD, and HET mice on HFD plus melatonin. β-actin was used as an internal control. The images are representative of three independent experiments. (HET): SIRT1+/- mice; (HFD): high fat diet (TD 03584-lard 35%); (MEL): melatonin; (STD): standard maintenance diet; (WT): wild type.


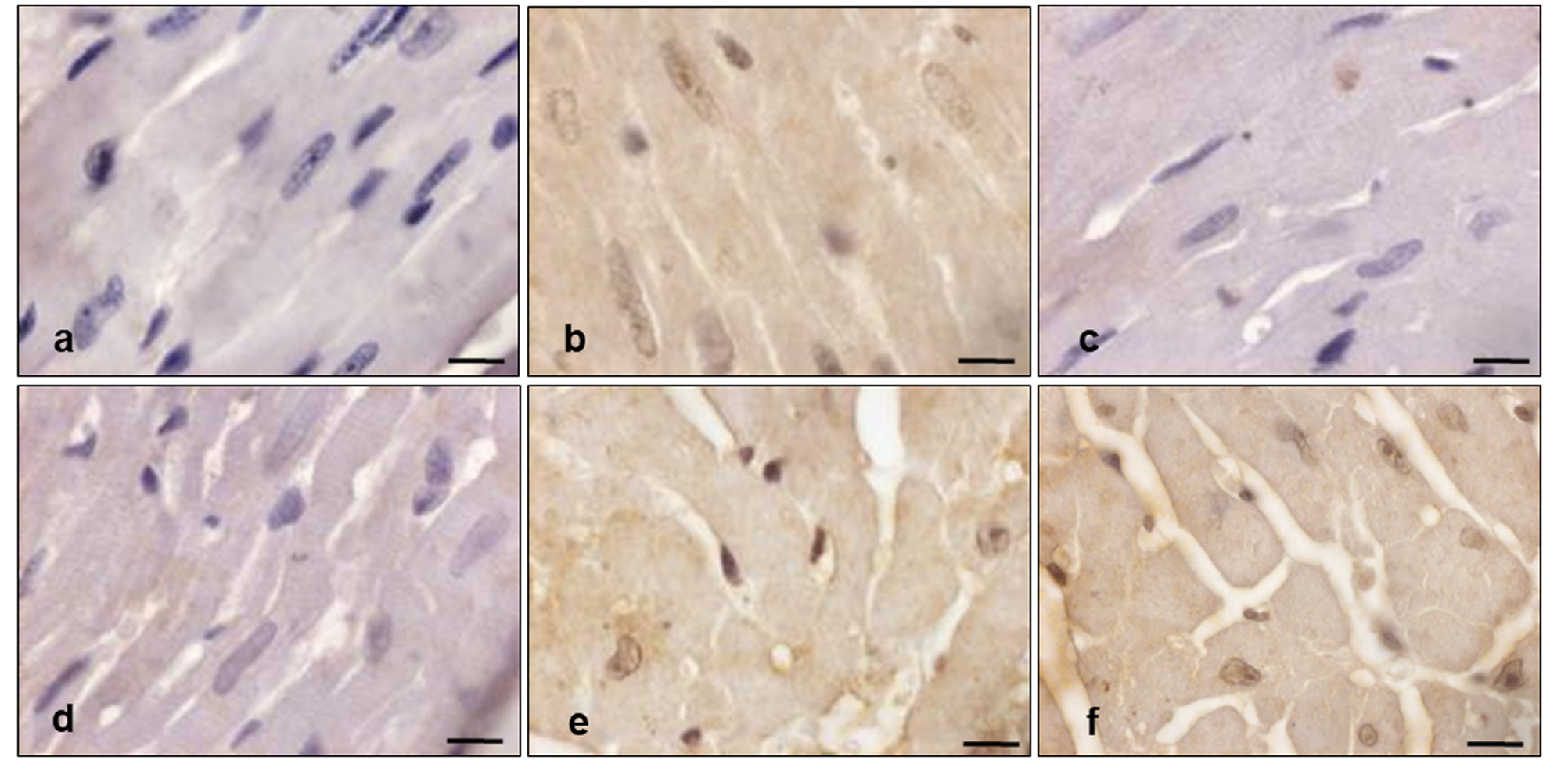


**Supplementary Figure S2**: *CHOP heart expression*. Representative pictures of nuclear proapoptotic CHOP staining in cardiomyocytes of WT on standard diet (**a**), WT mice on HFD (**b**), WT mice on HFD plus melatonin (**c**), HET mice on standard diet (**d**), HET mice on HFD (**e**), HET mice on HFD plus melatonin (**f**). Original magnification: 1000×; Bars = 10 µm.


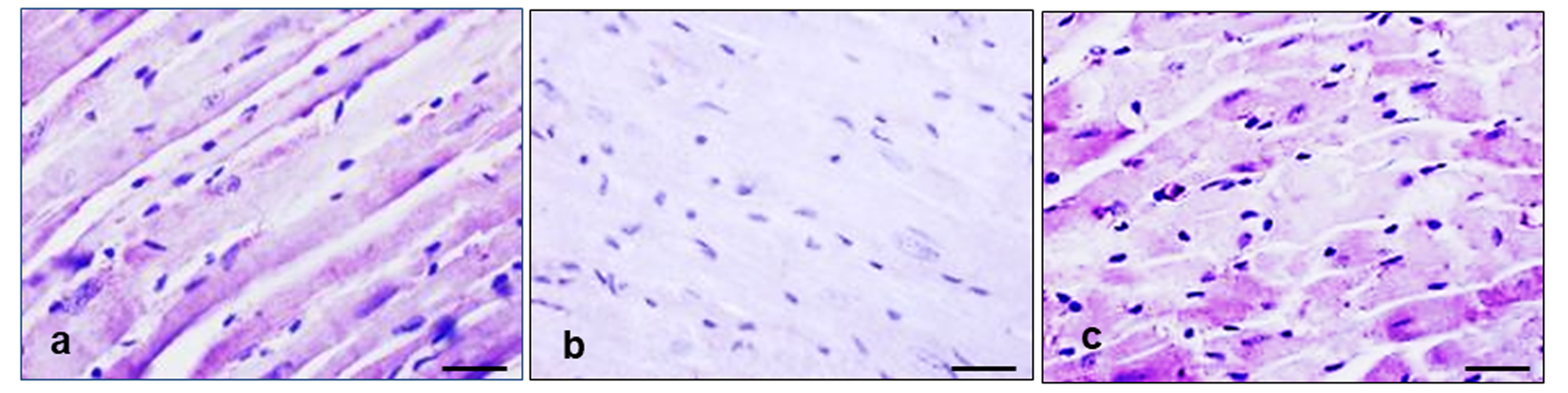


**Supplementary Figure S3:** *Mitofusin2 heart expression*. Representative pictures of mitofusin2 immunostaining of WT on standard diet (**a**), WT mice on HFD (**b**) and WT on HFD plus melatonin (**c**). Original magnification: 400×; Bars = 50 µm.


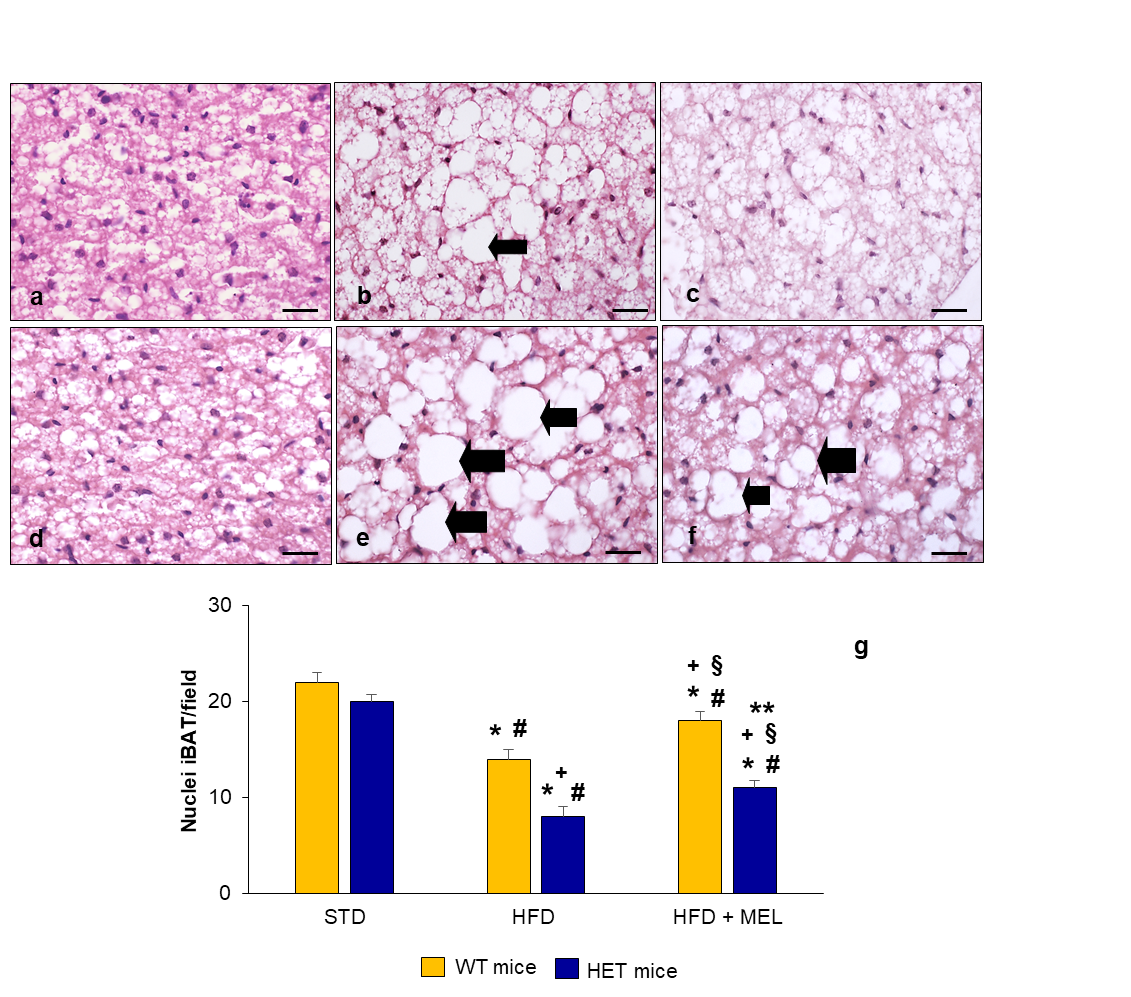


**Supplementary Figure S4**: *Interscapular brown adipose* *tissue (iBAT)* *histology.* Representative pictures of hematoxylin & eosin stained iBAT (**a**) WT mice on standard diet, (**b**) WT mice on HFD, (**c**) WT mice on HFD plus melatonin, (**d**) HET mice on standard diet, (**e**) HET mice on HFD, (**f**) HET mice on HFD plus melatonin. Black arrows indicate large lipid droplets in unilocular adipocytes. Original magnification: 400×. Bars= 20 µm. Quantitative analysis of the number of iBAT nuclei/field (g). * p< 0.05 vs WT mice on STD; # p<0.05 vs HET mice STD; + p< 0.05 vs WT mice HFD; § p< 0.05 vs HET mice on HFD; ** p< 0.05 vs WT mice on HFD plus melatonin. (iBAT): interscapular brown adipose tissue.
