# Supplementary material for: Cardiometabolic Changes in Sirtuin1-Heterozygous Mice on High-Fat Diet and Melatonin Supplementation
Source: Int J Mol Sci. 2024 Jan 10;25(2):860. doi: 10.3390/ijms25020860 (PMC10815439; doi:10.3390/ijms25020860)
Supplement: Supplementary file 1 [file ijms-25-00860-s001.zip › Supplementary Table S1.docx]

**Table S1.** Composition of standard rodent diet (A04-STD) and high fat diet (TD 03584-HFD) provided for 16 weeks.

| **%** | **A04-STD** | **TD 03584-HFD** |
| --- | --- | --- |
| Proteins | 19.2 | 15 |
| Lipids | 8.4 | 58.4 ^1^ |
| Carbohydrates | 72.4 | 26.6 |

^1^ Fat is composed by lard 35% weight-g/kg, containing cholesterol 0.95 mg/g, saturated fatty acids (palmitic, C16:0 and stearic acid, C18:0) and monounsaturated fatty acid (oleic acid C18:1). Total energy provided by HFD is 5.4 Kcal/g vs 3.15 Kcal/g by standard diet.
